# The use of a risk assessment and decision support tool (CRISP) compared with usual care in general practice to increase risk-stratified colorectal cancer screening: study protocol for a randomised controlled trial

**DOI:** 10.1186/s13063-018-2764-7

**Published:** 2018-07-25

**Authors:** Jennifer G. Walker, Finlay Macrae, Ingrid Winship, Jasmeen Oberoi, Sibel Saya, Shakira Milton, Adrian Bickerstaffe, James G. Dowty, Richard De Abreu Lourenço, Malcolm Clark, Louise Galloway, George Fishman, Fiona M. Walter, Louisa Flander, Patty Chondros, Driss Ait Ouakrim, Marie Pirotta, Lyndal Trevena, Mark A. Jenkins, Jon D. Emery

**Affiliations:** 1 Centre for Cancer Research, Department of General Practice, Victorian Comprehensive Cancer Centre, The University of Melbourne, Carlton, VIC Australia; 2 Department of Medicine, The University of Melbourne, Melbourne, VIC Australia; 3 Genetic Medicine and Family Cancer Clinic, Royal Melbourne Hospital, Melbourne, VIC Australia; 4 Colorectal Medicine and Genetics, Royal Melbourne Hospital, Melbourne, VIC Australia; 5 Centre for Epidemiology and Biostatistics, Melbourne School of Population and Global Health, The University of Melbourne, Melbourne, VIC Australia; 6 Centre for Health Economics Research and Evaluation, University of Technology Sydney, Sydney, NSW Australia; 7 IPN Medical Centres, Camberwell, VIC Australia; 8 Department of Health and Human Services, Victorian Government, Melbourne, VIC Australia; 9 Joint Consumer Advisory Group, Primary Care Collaborative Cancer Clinical Trials Group, Carlton, Australia; 10 The Primary Care Unit, Department of Public Health & Primary Care, University of Cambridge, Cambridge, UK; 11 Department of General Practice, The University of Melbourne, Melbourne, VIC Australia; 12 School of Public Health, The University of Sydney, Sydney, NSW Australia

**Keywords:** Colorectal cancer screening, Risk-stratified screening, Precision medicine, Primary care, Risk assessment tool, Decision support, Faecal occult blood test, General practice, Precision screening

## Abstract

**Background:**

Australia and New Zealand have the highest incidence rates of colorectal cancer worldwide. In Australia there is significant unwarranted variation in colorectal cancer screening due to low uptake of the immunochemical faecal occult blood test, poor identification of individuals at increased risk of colorectal cancer, and over-referral of individuals at average risk for colonoscopy. Our pre-trial research has developed a novel Colorectal cancer RISk Prediction (CRISP) tool, which could be used to implement precision screening in primary care. This paper describes the protocol for a phase II multi-site individually randomised controlled trial of the CRISP tool in primary care.

**Methods:**

This trial aims to test whether a standardised consultation using the CRISP tool in general practice (the CRISP intervention) increases risk-appropriate colorectal cancer screening compared to control participants who receive standardised information on cancer prevention. Patients between 50 and 74 years old, attending an appointment with their general practitioner for any reason, will be invited into the trial. A total of 732 participants will be randomised to intervention or control arms using a computer-generated allocation sequence stratified by general practice. The primary outcome (risk-appropriate screening at 12 months) will be measured using baseline data for colorectal cancer risk and objective health service data to measure screening behaviour. Secondary outcomes will include participant cancer risk perception, anxiety, cancer worry, screening intentions and health service utilisation measured at 1, 6 and 12 months post randomisation.

**Discussion:**

This trial tests a systematic approach to implementing risk-stratified colorectal cancer screening in primary care, based on an individual’s absolute risk, using a state-of-the-art risk assessment tool. Trial results will be reported in 2020.

**Trial registration:**

Australian and New Zealand Clinical Trial Registry, ACTRN12616001573448p. Registered on 14 November 2016.

**Electronic supplementary material:**

The online version of this article (10.1186/s13063-018-2764-7) contains supplementary material, which is available to authorized users.

## Background

Colorectal cancer is the third most common cancer in men (746,000 cases in 2012, 10.0% of the total cancer cases) and the second in women (614,000 cases in 2012, 9.2% of the total cases) worldwide. There is a wide geographical variation in incidence of colorectal cancer worldwide, with the highest rates in Australia and New Zealand and the lowest in Western Africa [1]. Internationally, screening is recognised as an effective method to reduce the burden of colorectal cancer. Randomised controlled trials of faecal occult blood test (FOBT) screening have demonstrated a 15–33% reduction in colorectal cancer mortality [2–4]. Despite this, few countries have an organised screening programme, and those with highly organised population-based screening often have a low screening uptake [5].

### Colorectal cancer screening in Australia

In Australia, biennial immunochemical FOBT (iFOBT) screening from age 50 years has been shown to be cost-effective with an estimated cost ranging from AUD$17,192 to 53,883 per year of life saved [6]. In 2006, the Australian government began the phased roll-out of a population-based colorectal cancer screening programme using the iFOBT. From 2020, the National Bowel Cancer Screening Program (NBCSP) will be fully implemented, offering biennial screening to all eligible people. The NBCSP sends a free iFOBT kit in the mail to all Australians between 50 and 74 years old for them to self-complete and return for analysis. Positive test results are managed by the participant’s nominated general practitioner who organises a colonoscopic investigation. Participation rates in the NBCSP remain low, with only 39% of the 2.6 million people invited returning a completed kit [7]. According to the Australian Commission on Safety and Quality in Healthcare report, almost 600,000 colonoscopies were performed in 2013–14 [8] suggesting that some people may be having colonoscopic screening instead of completing the NBCSP screening kit.

Australian guidelines (both previous and recently updated guidelines) recommend that colonoscopic screening should only be offered to individuals at increased risk on the basis of their family history of colorectal cancer [9, 10]. In 2012, based on the 2005 Australian Guidelines risk criteria [10], it was estimated that for every 1 million Australians 50 years and older, 80,000 people at average risk were being over-screened with colonoscopy, while 29,000 people at increased risk were not having the required colonoscopy [11, 12].

### Risk-stratified screening

People are not at equal risk of colorectal cancer. While the average population lifetime risk of colorectal cancer is around 5%, there is a wide spectrum of risk with large proportions of the population actually below or above the “average” risk [13].

Colorectal cancer risk for the quarter of the population with the highest contribution of risk factors is 20 times greater than for those in the lowest quartile, and 90% of colorectal cancer occurs in those in the upper half of the population for these risk factors [13]. Australian guidelines recommend screening with an iFOBT for people at “average and slightly increased risk” and colonoscopy for those at “increased risk”. However, these guidelines rely only on age and family history and are limited in their ability to detect individuals at high risk [10]. Risk prediction models can provide estimates of colorectal cancer risk so that more expensive and higher risk preventive strategies, such as colonoscopy, can be targeted at those most likely to benefit [14, 15].

### What is the difference between a cancer risk prediction model and a cancer risk assessment tool?

Risk prediction models are mathematical algorithms which combine demographic, clinical, lifestyle and genetic factors to accurately determine future risk of developing colorectal cancer [16]. A risk assessment tool applies a risk prediction model and presents risk information in a way that will change the actions of patients and clinicians. Identifying an accurate risk prediction model is simply the first step. Translating a risk model into clinical practice requires a risk assessment tool that is easy to use in a clinic, can improve practice and is acceptable to clinicians and patients.

An Australian colorectal cancer risk prediction model was developed and validated using data from the Colon Cancer Family Registry (CCFR) and includes risk/protective factors: age, sex, body mass index (BMI), diet (red meat and fruit), smoking (pack-years), previous colorectal cancer screening (FOBT and/or colonoscopy), polyp detection, use of medication (including non-steroidal anti-inflammatories, hormone replacement therapy, calcium) and first-degree relatives with colorectal/endometrial/ovarian cancer and age of diagnosis [14].

**Fig. 1:**
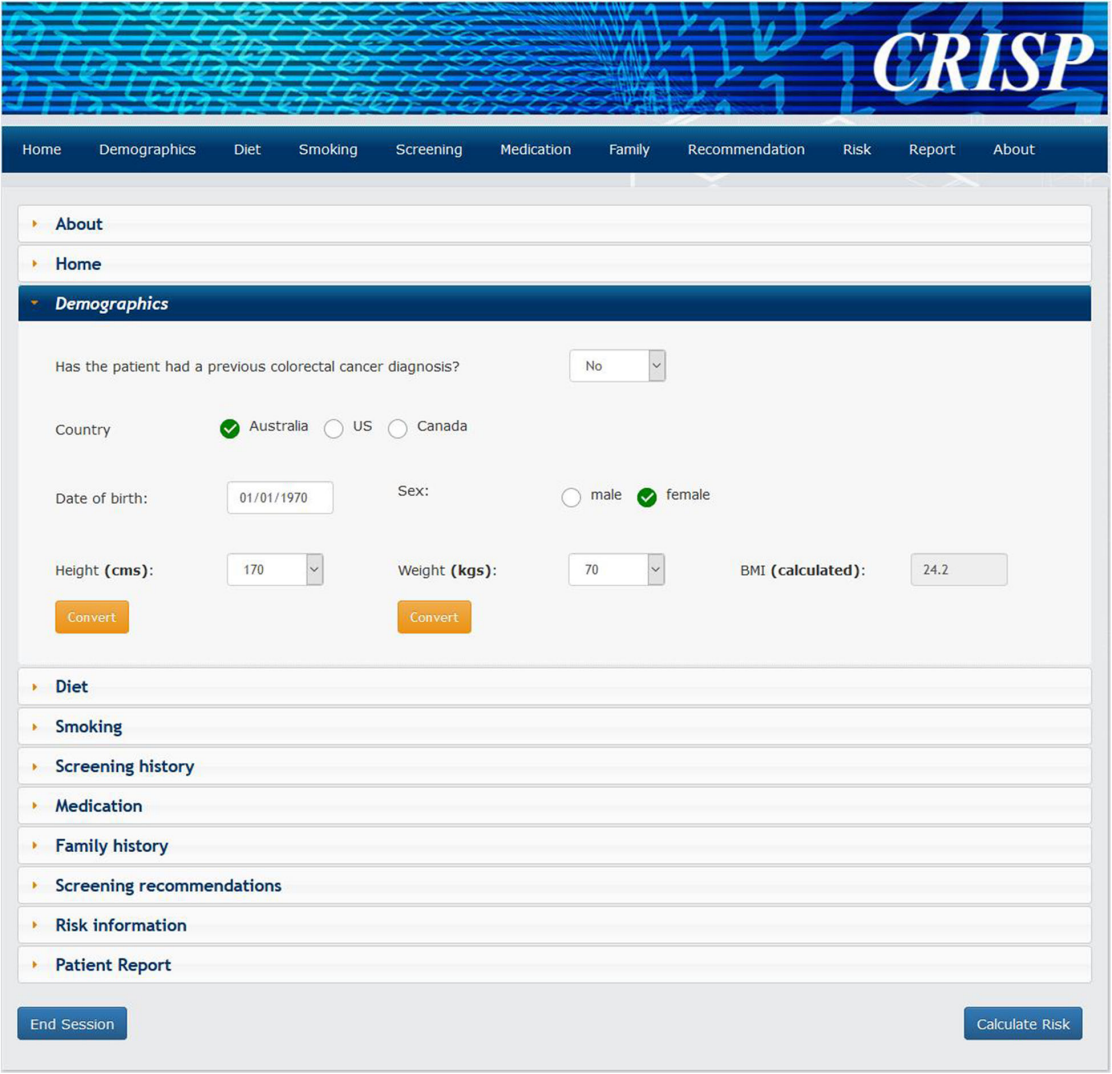
Screenshot of the CRISP tool with an example of a data entry screen. CRISP Colorectal cancer RISk Prediction

**Fig. 2:**
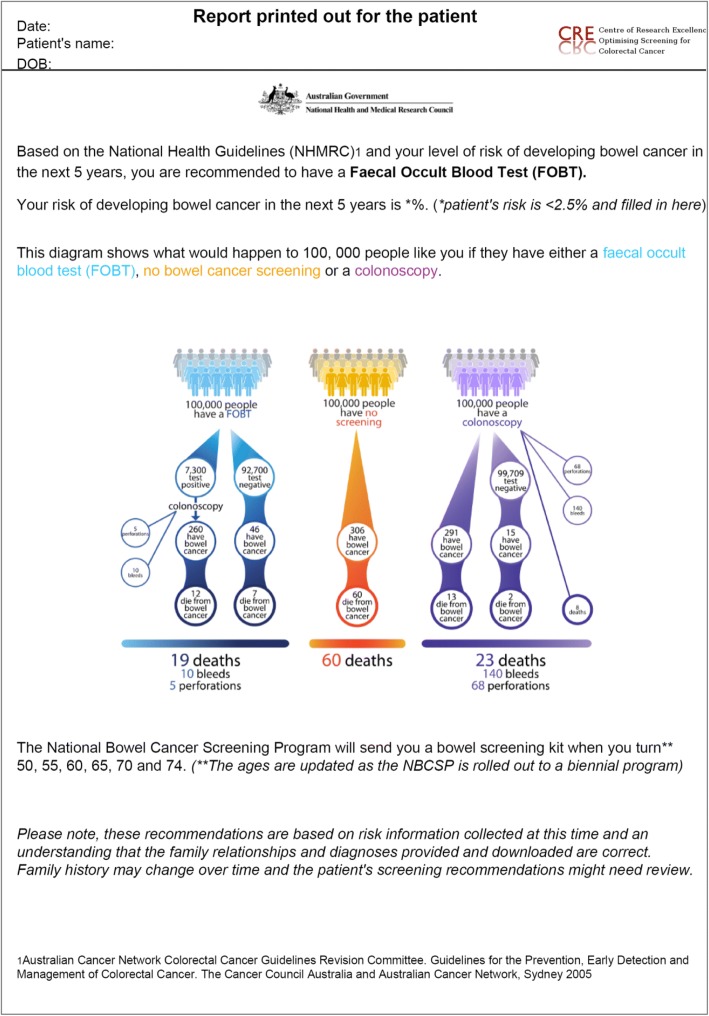
Example of a patient report generated by the CRISP tool

The Colorectal cancer RISk Predictor (CRISP) tool is a web-based risk assessment tool [17] which calculates an individual’s absolute risk of developing colorectal cancer (presented as 5-year and lifetime risk) (Fig. 1) based on an analysis of the Colon Cancer Family Registry [18]. The CRISP tool presents the risk information using previously evaluated risk communication formats [19, 20], and provides clinical decision support about recommended screening (Fig. 2). A 2.5% 5-year absolute risk of colorectal cancer was set as the threshold for switching from recommending biennial FOBT testing to 5-yearly colonoscopy. This threshold was chosen to be consistent with the risk categories recommending colonoscopy in the current National Health and Medical Research Council (NHMRC) guidelines. The CRISP tool also identifies people with a family history suggestive of a rare inherited cancer syndrome (e.g. Lynch syndrome) and recommends referral to a family cancer clinic rather than providing screening advice.

The development and evaluation of the CRISP tool has been informed by the UK’s Medical Research Council Framework for the design and evaluation of complex interventions [21, 22]. The CRISP tool design was informed by a qualitative study involving 14 GPs who used a prototype version of the CRISP tool in simulated consultations [17]. The methods to communicate risk information were tested using a randomised vignette study with patients in general practice. This informed a selection of expected frequency trees and comparative risk graphs as the primary method of risk communication for patients at average and increased risk respectively [20].

This protocol is for a multi-site, individually randomised controlled trial to test a primary care-led model of risk-stratified colorectal cancer screening, using the CRISP tool. The aim is to increase iFOBT uptake and reduce inappropriate colonoscopic screening in average-risk people, and to increase colonoscopic screening in people identified at higher risk. Our trial procedures were piloted in two general practices involving 85 participants. The pilot study demonstrated feasibility of the recruitment and randomisation procedures, refined the content of the CRISP consultation and confirmed acceptability of collecting patient-reported outcome measures [23].

The study protocol adheres to the SPIRIT statement (Additional file 1) [24].

### Objectives

The primary objective of the CRISP trial is to determine whether the effect of a standardised consultation using the CRISP risk assessment tool in general practice, compared with the provision of generic cancer prevention information, increases the proportion of participants who undergo risk-appropriate colorectal cancer screening; that is, completion of the right screening test (iFOBT or colonoscopy) based on an individual’s absolute risk of colorectal cancer and the Australian Guidelines at the time [10]. The secondary objectives are: (1) to test the CRISP tool compared with generic cancer risk reduction information on participants’ level of colorectal cancer risk perception, general anxiety, cancer worry and cancer screening intentions; and (2) to evaluate the CRISP tool on health service utilisation and healthcare costs.

### Hypotheses

1. A standardised consultation using the CRISP tool in general practice will increase risk-appropriate screening compared with generic information about cancer prevention at 12-month follow-up.
2. The CRISP tool will increase the accuracy of participants’ risk perception and their intentions for risk-appropriate screening without an adverse increase in cancer-specific anxiety after 1-month, 6-month and 12-month follow-up.

## Methods

### Participants, interventions and outcomes

#### Study setting

This is a multi-site individually randomised controlled trial (RCT) set in at least eight general practices (primary care practices) in Melbourne, Australia. General practices are purposively sampled from VicRen, the University of Melbourne practice-based research network which includes at least 200 general practices [25]. Practices are recruited from different areas in Melbourne to maximise the sociodemographic diversity of participants.

Participants are recruited from general practice waiting rooms prior to an appointment with their GP. General practices are eligible if they have at least 1000 active patients between 50 and 74 years old, at least three consenting doctors and a private room available for recruiting for at least 3 days per week.

Before starting recruitment in each general practice, the research team meet with the GPs and practice nurses to ensure they understand the expected clinical action in response to the CRISP tool-generated risk assessment reports, including how to respond to the participants in the control arm.

### Participants

Eligible participants for the trial are aged between 50 and 74 years old, which is consistent with the target population for the Australian NBCSP. Participants are able to read and write English and competent to give informed consent. Patients with a previous diagnosis of colorectal cancer are excluded as the CRISP tool is only designed for people who have never had colorectal cancer. Patients with recent rectal bleeding or inflammatory bowel disease are excluded and referred for assessment by their GP because they may require colonoscopy as a diagnostic rather than screening procedure. Patients with known Lynch syndrome, familial adenomatous polyposis or any other specific genetic predisposition for colorectal cancer, ascertained by a detailed family history of colorectal and other cancers, are excluded/family history of colorectal and other cancers, are excluded.

### Recruitment

**Fig. 3:**
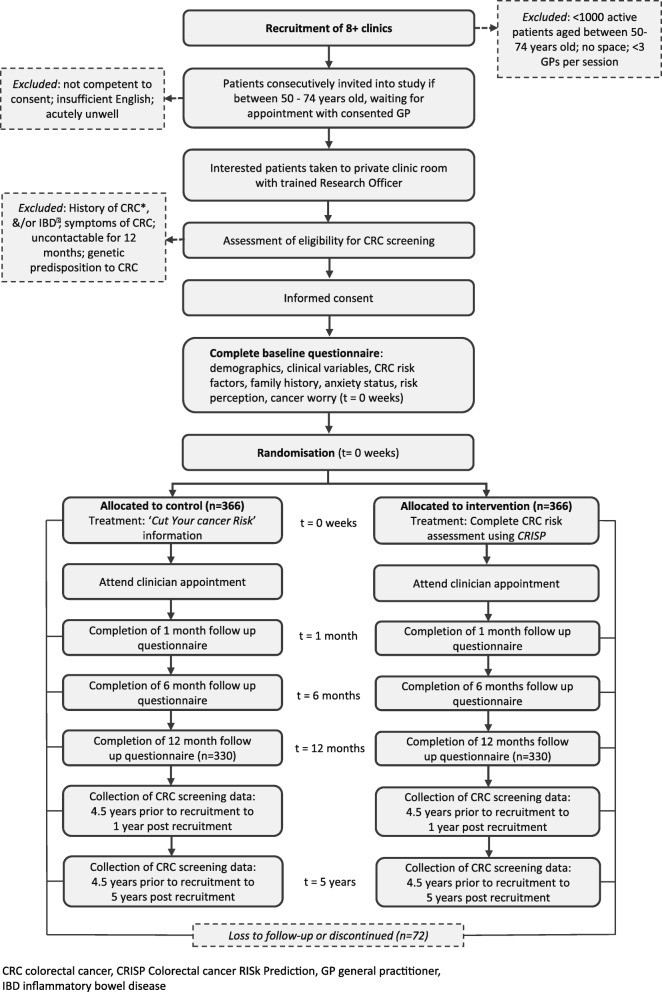
Trial flow chart (refer to Fig. 4 data collected at each time point)

Participants attending a GP consultation for any reason are recruited directly from the waiting room of general practices. Eligible patients between 50 and 74 years old, identified by reception staff from the daily appointment list, are approached consecutively in the waiting room by a research assistant (RA). If the patient is interested in the study and fulfils the initial criteria (age and appointment with a consenting GP), the RA takes them through to the CRISP research officer (RO) in a private room. The RO will discuss the trial and determine their eligibility. This method of recruitment and intervention delivery is designed to maximise accrual and reduce recruitment bias, a significant flaw in most of the previous trials of cancer risk assessment tools (Fig. 3) [26].

If the patient is eligible and interested, the RO explains the plain language statement and consents to the patient (Additional file 2). The participant is given a signed copy of the consent form for his/her records. The RO completes an online baseline questionnaire with the participant once he/she has consented to the study but prior to randomisation. The baseline questionnaire captures a specific clinic identification code and other response data, and these data are embedded into a clickable link at the conclusion of the survey. This link points to another server which hosts randomisation software. Randomisation is automated on completion of the online baseline questionnaire.

### Intervention arm

Delivery of the intervention occurs on the day of recruitment prior to the participant’s consultation with their GP. After completion of baseline data collection, those randomised to the intervention arm have their baseline data automatically uploaded into the CRISP tool. Using the risk calculation and risk-specific screening recommendations generated by the CRISP tool, the RO discusses the participant’s risk of colorectal cancer and recommendations about appropriate colorectal cancer screening using a standardised consultation script. A print-out summarising the participant’s colorectal cancer screening recommendations and risk is given to them to discuss with their GP (Fig. 2). The CRISP tool also generates a report for the GP which is subsequently uploaded into the participant’s electronic records. If the participant is classified by the CRISP tool to be at average risk and is due for an iFOBT (i.e. has not completed one in the previous 2 years), he/she is provided with a free iFOBT kit and asked to discuss this with his/her GP. The RO provides brief advice on how to complete the iFOBT kit. Those at increased risk who require a referral for colonoscopy are advised to discuss this with their GP. Participants who are given an iFOBT kit during the CRISP consultation receive a reminder by text message at 1 month to prompt them to complete their kit. The intervention arm participants also receive generic information on how they can reduce their colorectal cancer risk.

The core intervention is the CRISP consultation but the other components are designed to increase self-efficacy to complete the FOBT for those at average risk as part of an overall complex intervention [21, 22].

### Control arm

After completion of the online baseline data, participants randomised to the control arm are automatically directed to an electronic presentation of the Cancer Council Victoria’s *Cut Your Cancer Risk* brochure [27]. The RO discusses this with the participant using a standardised consultation script. The focus of the consultation involves explaining modifiable factors that can reduce the cancer risk including lifestyle changes, and brief information about the national cancer screening programmes for breast, cervical and colorectal cancer. This is designed as a credible attention control which also increases engagement in the trial for control participants to minimise attrition. All participants receive a hard copy of the brochure. Participants randomised to the control arm continue to access health services as “usual care”.

### Random allocation, concealment mechanism and blinding

Random allocation of the participant to either the intervention or control arm is automated on completion of the online baseline questionnaire to ensure allocation concealment. The random allocation sequence, stratified by general practice, was computer-generated by our statistician (PC) with a 1:1 allocation ratio using random permuted block sizes of four, six and eight within each stratum. This sequence is incorporated into the randomisation software. Within each clinic (stratum), the randomisation software allocates the randomisation code sequentially and then redirects the browser to the CRISP Web App if the participant is randomised to the intervention arm, or to a series of screens with the Cancer Council Victoria’s *Cut Your Cancer Risk* information [27] if randomised to the control arm.

### Data collected

Baseline data collected include: demographics (age, sex, postcode, language spoken at home and country of birth, marital status, highest education level attained) and clinical variables (age, height, weight, number of first-degree relatives with colorectal cancer and ages of diagnoses, smoking history, current fruit and red meat consumption, history of use of non-steroidal anti-inflammatory drugs, current use of hormone replacement therapy (women only) and calcium supplements, and history and clinical outcomes of previous FOBT, colonoscopy including previous polyps) [15]. Additional baseline measures of CRC risk perception [28, 29], generalised anxiety [30], cancer worry [31, 32] and intention to screen [33] are also included in the baseline questionnaire.

Health service use is tracked using data extracted from the Australian Government Department of Health Medicare Benefits Schedule (MBS) [34] that maintains information about visits to healthcare providers and diagnostic tests, the National Bowel Cancer Screening data to access FOBT screening and the Victorian Hospital Admitted Episodes Dataset (VAED) to access privately funded colonoscopic screening.

### Outcome measures

The primary outcome is the proportion of participants who have had risk-appropriate colorectal cancer screening measured at 12-month follow-up. For participants in the intervention arm, appropriateness of screening will be determined by the CRISP tool and is based on the participant’s 5-year absolute risk of colorectal cancer, using the 2.5% threshold, and their concordance with the recommended mode and frequency of screening. For the participants in the control arm, family history and previous screening data collected in the baseline questionnaire will be used to determine risk-appropriate screening in accordance with National Guidelines at the time of recruitment.

The CRISP tool was developed using the previous 2005 Australian guidelines which were revised in October 2017. As our trial was already underway with more than 300 participants recruited, the Trial Steering Committee agreed that it was not appropriate to change the recommendations halfway through recruitment. Nonetheless, the changes to the risk criteria in the revised guidelines were relatively minor but their potential impact will be examined in a pre-planned sensitivity analysis (see later) [9].

Screening behaviour will be obtained for all of the participants from the following sources: participant self-report; data from GP records (results of FOBT and/or colonoscopy); Medicare Benefits Schedule through the Department of Human Services (for specific colonoscopy item numbers); the National Bowel Cancer Screening Program; and the Victorian Admitted Episodes Dataset (for colonoscopies conducted in Victorian public hospitals not captured by MBS data).

The data collection will be done systematically and we will use a hierarchical approach to determine the final screening behaviour with self-report at the bottom of the hierarchy. Where possible, self-report will be validated from other data sources. The Steering Committee have formed a clinical sub-committee to make decisions about “appropriate screening” behaviour for discordant reported screening behaviour from the multiple data sources. Blinded clinical review will be conducted on a case-by-case basis to reach consensus when there are discordant results.

### Secondary outcomes measured at 1, 6 and 12 months

1. Risk perception: family history of colorectal and other cancers, are excluded perceived risk, both absolute and comparative, is measured using validated scales from our previously published systematic reviews and primary research on colorectal cancer risk [28, 29].
2. Generalised anxiety using the State-Trait Anxiety Inventory (STAI) scale [30].
3. Cancer-specific anxiety using an established measure applied extensively in cancer screening research, and previously modified for colorectal cancer [31, 32].
4. Intentions to have an iFOBT and/or colonoscopy in the next 3 months, based on items from the Theory of Planned Behaviour and previous research [29, 33].
5. Clinical outcomes of screening tests (e.g. detection of polyps and cancers), obtained from GP records (Additional file 3).

### Other secondary outcomes

6. Proportion of participants who have had risk-appropriate screening measured after 5 years from baseline.
7. Health service utilisation and healthcare costs resulting from implementation of the CRISP tool.

### Measurement timing

Colorectal cancer screening behaviour will be collected for a maximum period of 4.5 years prior to recruitment (the maximum period for which historic Medicare Benefits Schedule data can be requested) and 5 years from recruitment. The timing, modality and results of any previous screening will be used to determine the time interval and modality of screening during the trial period to measure the appropriate screening outcome. Screening data at 1 year after recruitment will be used to determine the primary outcome.

**Fig. 4:**
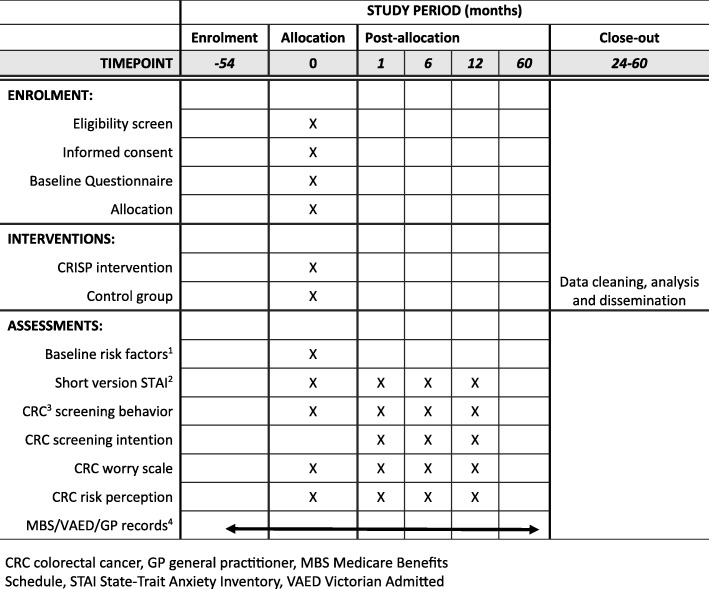
Schedule of enrolment, interventions and assessments. Baseline risk factors include age, sex, height, weight, smoking, medications, dietary habits and previous colorectal cancer screening

The participant-completed outcome measures (self-reported screening behaviour, risk perception, general anxiety, cancer worry and intention to screen) are captured at baseline and 1, 6 and 12 months (Fig. 4).

### Sample size and power calculation

Sample size calculations were initially based on previously published rates of risk-appropriate screening [11, 12] and a 6.5% prevalence of increased risk of colorectal cancer in a primary care population [35]. Based on these data, allowing for 10% attrition at 12-month follow-up, to achieve 90% power with a two-sided 5% level of significance, the sample size was calculated at 278 participants per arm to detect, at a minimum, a 10% difference in the percentage of risk-appropriate screening at 12 months between the intervention and control arms. Given the low estimated rates of risk-appropriate screening, we considered several scenarios where the risk-appropriate screening at 12 months also increased in the control arm (Table 1) and selected a more conservative sample size that was achievable and practical. Using data from the NBCSP [36] and data on the benefits and harms of colonoscopy [5, 37], an estimated 10% improvement in risk-appropriate screening would be associated with the following outcomes per 1 million people aged 50 and older: 92,000 more faecal occult blood tests, 3000 fewer colonoscopies and 55 fewer deaths.

**Table 1:** Required samples sizes for three different scenarios of increases in risk-appropriate screening in the intervention arm compared to the control arm, assuming rate of risk-appropriate screening of 1% and 25% at baseline and level of significance = 0.05

Scenario (intervention vs control)	Absolute difference in appropriate screening	Power	Sample size per arm	Sample size per arm allowing for 10% attrition
10% improvement vs 0%	11% vs 1%	90%	117	130
10% improvement vs 2.5%	11% vs 3.5%	90%	250	278
10% improvement vs 5%	11% vs 6%	90%	652	725
10% improvement vs 0%	35% vs 25%	80%	329	366

### Revised sample size: updated using baseline data

Recruitment started in May 2017 after ethics approval was granted. In December 2017 it became clear that we were recruiting faster than anticipated and were close to reaching our initial target sample size. At the quarterly Trial Steering Committee meeting it was decided to review the sample size assumptions to ensure that the study was adequately powered. We were concerned that the percentage of individuals with risk-appropriate screening may be higher in the primary care population than had been found in previous community-based studies [11, 12] that had been conducted prior to the roll-out of the NBCSP in 2006. Based on baseline survey and self-reported colorectal cancer screening of the 397 participants collected prior to randomisation, we conservatively estimated that risk-appropriate screening of the trial population at baseline was no greater than 25%. The Trial Steering Committee reviewed these data in February 2018 and recommended to increase the sample size to 366 per arm (allowing for 10% attrition over the 12 months) to ensure that the study has at least 80% power to detect a minimum of 10% difference in the percentage of risk-appropriate screening at 12 months between the intervention and control arms, assuming that 25% of the control participants receive risk-appropriate screening at 12 months.

### Statistical methods

Baseline participant characteristics will be compared between the two study arms using descriptive statistics to assess for chance imbalance. The primary analysis will compare the proportion of participants who have had risk-appropriate colorectal cancer screening at 12 months using logistic regression with the randomisation stratification factor, general practice, as a covariate. The analysis will be repeated for the risk-appropriate colorectal cancer screening at 5 years as a secondary longer-term outcome. Comparisons between arms on continuous secondary outcomes will use a linear mixed-effects model that includes arm (intervention and control), general practice and time (baseline, 1, 6 and 12 months) as fixed effects and individuals treated as random effects, with two-way interactions between study arm and time, except baseline where study arm means will be constrained to be equal.

Comparisons between study arms on binary secondary endpoints with repeated outcomes measures will be performed using logistic regression, using generalised estimating equations with robust standard errors to allow for the repeated outcome measures on individuals, with general practice included as a covariate. All analyses will be conducted in Stata 15 [38]. The estimated intervention effects will be reported as the odds ratio for binary outcomes and the difference in means between the intervention and control arms for continuous outcomes. All estimates will be reported with respective 95% confidence intervals and *p* values. For the secondary continuous outcomes, pre-specified adjustment for baseline outcome measure will increase the precision of the estimated intervention effect and may adjust the intervention effect if there is imbalance on this measure at baseline.

A sensitivity analysis will be performed on the primary and secondary endpoints to adjust for additional pre-specified baseline variables, for example age, sex and family history of colorectal cancer, in the regression models to explore whether the estimated intervention effect is affected by the potential for imbalance of these confounders in the sample.

A sensitivity analysis will also be conducted to assess the impact of using the revised 2017 Australian Colorectal Screening guidelines on the proportion of participants classified as having risk-appropriate colorectal cancer screening at 12 months and 5 years. All randomised participants will be included in the analysis in their assigned study arms in accordance with the intention-to-treat principle [39]. Possible differential attrition will be assessed by comparing baseline characteristics of those who withdraw against those who remain in the study. Appropriate methods for dealing with missing data will be detailed in the statistical analysis plan and be informed by a blinded review of the data. Sensitivity analysis will be used to assess the robustness of the missing data assumption.

A health economic analysis will combine a within-trial and modelled analysis of the cost-effectiveness of implementing the CRISP tool. The within-trial analysis will focus on the incremental cost per appropriately screened individual for the CRISP tool versus usual care. The modelled analysis will combine observed colorectal cancer detection rates from the trial with data from the published literature to investigate the longer-term cost-effectiveness of the CRISP tool using an economic model. Within-trial costs in the two study arms will be estimated based on data observed from general practice, MBS, the NBCSP and the VAED records. Mean estimates of costs will be used and confidence intervals will be generated by resampling (boot-strap) techniques. Benefits in the modelled analysis will be assessed as quality-adjusted life years (QALYs) by extrapolating the observed cancer detection rates from the trial to longer-term survival and adjusting them by published quality of life (utility) weights. Results will be presented in terms of the incremental cost-effectiveness ratio (ICER) as a cost per case detected for the within-trial period, and a cost/QALY gained for the modelled analysis. Sensitivity analyses will be conducted to test the robustness of the results of the economic analysis to variations in the underlying assumptions and inputs.

### Data monitoring

Participant information, including their preferred method for follow-up contact, will be automatically populated into Research Electronic Data Capture (REDCap) data management software from the baseline online questionnaire [40]. Follow-up questionnaires will be completed either via an emailed link to an online questionnaire, a paper questionnaire sent to the participant or a computer-assisted telephone interview. If surveys are not completed/returned within 2 weeks of being sent, a research assistant blinded to study arm allocation will telephone participants to remind them to complete the survey and offer to complete the survey over the phone. Participants will be contacted a maximum of three times by telephone to complete the survey. All information and data will be stored in password-protected computers only accessible to the study team.

The CRISP trial data will be monitored by a dedicated (and blinded) research assistant and overseen by the Steering Committee and led by the Chief Investigator (JDE). The Trial Steering Committee includes primary care experts, gastroenterologists, epidemiologists, senior research staff and a consumer who will meet quarterly to monitor recruitment progress, address any problems and ensure that the project is being conducted according to protocol. The research assistant will monitor trial processes and report complaints, harms and adverse events to the Trial Steering Committee. Given that the intervention is evidence based and participants are required to be under the duty of care of their GP, any adverse events will be reported to the GP with the participant’s permission. The most likely adverse event is increase in cancer worry which will be monitored weekly by the research assistant responsible for data monitoring. If any participant answers “often” for at least three of the six questions in the cancer worry scale questions or “almost all the time” for at least one of the cancer worry scale questions, they will be contacted by the research assistant who will advise them to follow this up with their GP if they have ongoing concerns. Any adverse events will be recorded (including relation to study, severity, potential for the event to have been anticipated and action taken) and reported to the Trial Steering Committee. Serious adverse events will also be reported to the university ethics committee.

### Minimising contamination

To maximise the fidelity of the methods, ROs have been trained using simulated consultations to deliver both the CRISP and control consultations in a standardised way. Specific consultation scripts for the intervention and control arms are used to minimise the risk of contamination. A random 10% of trial consultations is monitored through audiotaping and reviewed by the research team to ensure fidelity to the consultation scripts. To reduce participant contamination bias, only one participant per household will be invited into the trial (if known). To reduce clinician contamination, GPs are advised of the trial protocols, including having written flow charts to remind them of their expected management of intervention and control participants. GPs will not have access to the CRISP tool.

There is a small risk that GPs’ awareness of colorectal cancer screening will be raised during the trial, which could alter their total number of referrals for iFOBT and colonoscopy, but not on the risk-appropriateness of these referrals. Control participant GP records will be evaluated to measure the proportion of iFOBTs ordered by the GP within 2 weeks of recruitment as a proxy measure of contamination.

### Blinding

The nature of the intervention means that participants cannot be blinded to their treatment allocation. GPs will only be aware of participants allocated to the intervention arm and will not be informed when a participant is allocated to the control arm. The majority of follow-up questionnaires will be completed by participants online or via post and those completed by telephone are done so with a blinded researcher. For the extraction of health service utilisation data, research staff will be blinded to allocation assignment. All study analyses will be conducted by a statistician blind to the participants’ study arm allocation; study arm allocation will be coded as A or B when data are extracted from the online databases and supplied to the statistician, with the key for the intervention or control arm revealed after data are analysed and interpreted. Investigators will also remain blind to the study arm allocation when interpreting the results.

### Process evaluation

The quantitative trial data will be supplemented by qualitative semi-structured, in-depth interviews after 12 months to assess the impact and experience of undergoing colorectal cancer risk assessment in primary care. A purposive sample of participants will be interviewed, comprising people at increased and average risk in the intervention arm with a range of age and gender. The sample will include participants whose screening intentions and behaviours were changed and participants whose screening intentions remained unaltered by the CRISP intervention. Approximately 20 people will be sufficient to generate a full range of themes and perspectives, and allow for theme saturation in key areas. Interviews will be undertaken by an experienced qualitative researcher, and will be informed by a topic guide based on relevant literature and revised based on emerging findings from the iterative analytic process [35, 41].

### Ethics and dissemination

The study protocol has been approved by The University of Melbourne Human Research Ethics Committee (HREC ID number 1647804). The Australian Department of Human Services (Reference Number MI6113) approved the collection of Medicare Benefits Scheme and health service utilisation data for the purpose of colorectal cancer screening, and the Victorian Department of Health and Human Services approved collection of VAED data for the same purposes. Important protocol changes will be reported to the trial register and ethics committees as necessary. Eligible patients will be provided with a plain language statement outlining important information about the study in addition to receiving a signed copy of the consent form.

Each participant will be given a unique identification number and all information provided by them will remain confidential. All data are stored securely on University of Melbourne password-protected computers within locked facilities as required by the University of Melbourne Ethics Committee. Only investigators included in the original ethics applications or subsequent amendments have access to the identified dataset.

The results of the study will be presented at relevant conferences and published in peer-reviewed journals as per the CONSORT guidelines. The trial findings will be disseminated to participants who have indicated their wish to be informed of the results of the study. GP clinics that have participated in the trial will be provided with a community report and presented with the results at a clinic meeting. The findings will be presented at international meetings including those of the International Cancer in Primary Care (Ca-PRI meeting) and the Society for Academic Primary Care (UK), and also to contributing research institutions including the Universities of Cambridge (UK) and Washington (St Louis, MO, USA). The data including the statistical code will not be available for public access.

## Discussion

### Pragmatic vs explanatory trials

The PRECIS framework recognises the continuum between pragmatic and explanatory trials [42]. Pragmatic (or phase III) trials test interventions in real-world settings and often represent the final phase of trialling an intervention. Explanatory trials (also called phase II or efficacy trials) are more appropriate for novel interventions and aim to test whether an intervention, delivered in an ideal way, might work. The CRISP tool is being tested in an ideal way using trained researchers and comparing it against a standardised comparison with the understanding that if the CRISP tool cannot alter colorectal cancer screening when applied in this ideal way, then there is no chance of it working in the real world. In the past, there have been many pragmatic trials in primary care with negative findings conducted without a prior phase II trial. These negative pragmatic trials cannot say whether it was because the intervention itself was poorly designed, whether it was poorly implemented or whether the trial was poorly conducted. This is the rationale for a phase II trial of this novel intervention. If the CRISP tool demonstrates a moderate effect, this will justify proceeding to a larger phase III pragmatic cluster randomised trial. If the tool demonstrates a large beneficial effect, the trial will inform a widespread implementation strategy instead [21].

### Individual vs cluster randomised controlled trials

The relative advantages and disadvantages of participant-level versus practice-level randomisation were carefully considered when planning this trial. Our systematic review of previous RCTs of cancer risk assessment tools [26] highlighted the major disadvantages of cluster randomised trials, including recruitment bias and the inability to obtain participant-reported outcomes in control practices [32] and the significant additional costs of recruiting sufficient practices to achieve power [43]. Based on previous primary care research in a lung cancer screening study (CHEST Trial), individual participant-level randomisation was chosen [44].

Colorectal cancer is a major health problem for the western world. Early detection through faecal occult blood screening is a highly cost-effective strategy [6].

This trial tests a systematic approach to implementing colorectal cancer screening in primary care so that the right person receives the right test at the right time. More significantly, it will test a novel and transformative approach to precision cancer screening in primary care, based on an individual’s absolute risk of a specific cancer, using a state-of-the-art risk assessment tool. The trial findings will be reported in late 2020.

## Trial status

Protocol version 3 (27 September 2017); recruitment start date 9 May 2017; projected recruitment completion date 3 July 2018.

## Additional files

Additional file 1: SPIRIT checklist. (DOC 135 kb)

Additional file 2: Informed consent materials. Plain language statements and consent forms. (PDF 376 kb)

Additional file 3: Additional outcome variables. (DOCX 17 kb)

## Abbreviations

BMI: Body mass index
CCFR: Colon Cancer Family Register
CRC: Colorectal cancer
CRISP: Colorectal cancer RISk Prediction
FOBT: Faecal occult blood test
GP: General practitioner (primary care physician)
MBS: Medicare Benefits Schedule
NBCSP: National Bowel Cancer Screening Program (Australia)
NHMRC: National Health and Medical Research Council (Australia)
PRECIS: Pragmatic-Explanatory Continuum Indicator Summary
RCT: Randomised controlled trial
RO: Research officer
VAED: Victorian Admitted Episodes Dataset (hospital admissions)

## References

[1] Ferlay J, Soerjomataram I, Ervik M, Dikshit R, Eser S, Mathers C, et al. GLOBOCAN 2012 v1.0, Cancer Incidence and Mortality Worldwide: IARC CancerBase No. 11 [online]. Lyon, France: IARC, WHO; 2013. http://globocan.iarc.fr. Date cited: 2017 Aug 18.

[2] Hardcastle JD, Chamberlain JO, Robinson MH, Moss SM, Amar SS, Balfour TW (1996). Randomised controlled trial of faecal-occult-blood screening for colorectal cancer. Lancet.

[3] Mandel JS, Church TR, Bond JH, Ederer F, Geisser MS, Mongin SJ (2000). The effect of fecal occult-blood screening on the incidence of colorectal cancer. N Engl J Med.

[4] Zheng S, Chen K, Liu X, Ma X, Yu H, Chen K (2003). Cluster randomization trial of sequence mass screening for colorectal cancer. Dis Colon Rectum.

[5] Schreuders EH, Ruco A, Rabeneck L, Schoen RE, Sung JJY, Young GP (2015). Colorectal cancer screening: a global overview of existing programmes. Gut.

[6] Pignone M, Flitcroft K, Howard K, Trevena L, Salkeld G, St John D (2011). Costs and cost-effectiveness of full implementation of a biennial faecal occult blood test screening program for bowel cancer in Australia. Med J Aust.

[7] Australian Institute of Health and Welfare. National Bowel Cancer Screening Program: monitoring report 2017. Canberra: Australian Government; 2017.

[8] Australian Commission on Safety and Quality in Healthcare. Australian Atlas of Healthcare Variation. Sydney: Australian Government; 2015.

[9] Clinical practice guidelines for the prevention, early detection and management of colorectal cancer—Cancer Guidelines Wiki [Internet]. 2017 [cited 2017 Nov 11]. Available from: http://wiki.cancer.org.au/australia/Guidelines:Colorectal_cancer.

[10] Australian Cancer Network Colorectal Cancer Guidelines Revision Committee (2005). Guidelines for the Prevention, Early Detection and Management of Colorectal Cancer.

[11] Ait Ouakrim D, Boussioutas A, Lockett T, Winship I, Giles GG, Flander LB (2012). Screening practices of unaffected people at familial risk of colorectal cancer. Cancer Prev Res.

[12] Ait Ouakrim D, Lockett T, Boussioutas A, Keogh L, Flander LB, Winship I, et al. Screening practices of Australian men and women categorized as “at or slightly above average risk” of colorectal cancer. Cancer Causes Control. 2012;23(11):1853–64. 10.1007/s10552-012-0067-y PMC3508400 23011536

[13] Hopper JL. Disease-specific prospective family study cohorts enriched for familial risk. Epidemiol Perspect Innov. 2011;8(1):2. [cited 2017 Aug 22]. Available from: https://epi-perspectives.biomedcentral.com/articles/10.1186/1742-5573-8-2. 10.1186/1742-5573-8-2 PMC3055804 21352566

[14] Zheng Y, Hua X, Win A, Jenkins M, Macinnis R. Abstract PR05: Does a comprehensive family history of colorectal cancer improve risk prediction? In: Improving Cancer Risk Prediction for Prevention and Early Detection [Internet]. Orlando: AACR; 2017. [cited 2017 Jul 5]. Available from: http://cebp.aacrjournals.org/content/26/5_Supplement/PR05.abstract.

[15] Jenkins MA, Makalic E, Dowty JG, Schmidt DF, Dite GS, MacInnis RJ (2016). Quantifying the utility of single nucleotide polymorphisms to guide colorectal cancer screening. Future Oncol.

[16] Usher-Smith J, Emery JD, Hamilton W, Griffin SJ, Walter FM. Risk prediction tools for cancer in primary care. Br J Cancer. 2015;1(12)1645–50. 10.1038/bjc.2015.409. Epub 2015 Dec 3. 10.1038/bjc.2015.409 PMC4701999 26633558

[17] Walker JG, Bickerstaffe A, Hewabandu N, Maddumarachchi S, Dowty JG, CRECRC (2017). The CRISP colorectal cancer risk prediction tool: an exploratory study using simulated consultations in Australian primary care. BMC Med Inform Decis Mak.

[18] Jenkins MA, Win AK, Templeton AS, Angelakos MS, Buchanan DD, Cotterchio M, et al. Cohort profile: the Colon Cancer Family Registry Cohort (CCFRC). Int J Epidemiol. 2018;47(2):387–88i. [cited 2018 Mar 20]. Available from: https://academic.oup.com/ije/advance-article/doi/10.1093/ije/dyy006/4911903. 10.1093/ije/dyy006 PMC5913593 29490034

[19] Trevena LJ, Zikmund-Fisher BJ, Edwards A, Gaissmaier W, Galesic M, Han PKJ (2013). Presenting quantitative information about decision outcomes: a risk communication primer for patient decision aid developers. BMC Med Inform Decis Mak.

[20] Kim G, Walker J, Bickerstaffe A, Hewabandu N, Maddumarachchi S, Jenkins M (2018). The CRISP-Q study: communicating the risks and benefits of colorectal cancer screening. Aust J Gen Pract.

[21] Campbell M, Fitzpatrick R, Haines A, Kinmonth AL, Sandercock P, Spiegelhalter D (2000). Framework for design and evaluation of complex interventions to improve health. Br Med J.

[22] Craig P, Dieppe P, Macintyre S, Michie S, Nazareth I, Petticrew M (2008). Developing and evaluating complex interventions: the new Medical Research Council guidance. BMJ.

[23] Walker J, Bickerstaffe A, Hewabandu N, Saya S, Jenkins M, Emery J. A PHASE II trial exploring the feasibility of proposed methods for a large trial of a colorectal cancer risk prediction tool [CRISP]. In: Primary Care Cancer Clinical Trials Group (PC4) Conference. Melbourne: PC4; 2017.

[24] Chan A-W, Tetzlaff JM, Altman DG, Laupacis A, Gøtzsche PC, Krleža-Jerić K (2013). SPIRIT 2013 statement: defining standard protocol items for clinical trials. Ann Intern Med.

[25] Soós M, Temple-Smith M, Gunn J, Johnston-Ata’ata K, Pirotta M. Establishing the Victorian Primary Care Practice Based Research Network. Repr from Aust Fam PhysiciAn. 2010;39(11) [cited 2017 Dec 22]. Available from: https://www.researchgate.net/profile/Meredith_Temple-Smith/publication/49817811_Establishing_the_Victorian_Primary_Care_Practice_Based_Research_Network/links/0912f50644b1011bdb000000.pdf 21301660

[26] Walker JG, Licqurish S, Chiang PPC, Pirotta M, Emery JD (2015). Cancer risk assessment tools in primary care: a systematic review of randomized controlled trials. Ann Fam Med.

[27] Cancer Council Victoria. Cut your cancer risk [Internet]. 2017. Available from: http://www.cancervic.org.au/downloads/cpc/cut_your_cancer_risk_infosheet.pdf. Accessed Aug 2017.

[28] Braithwaite D, Emery J, Walter F, Prevost AT, Sutton S (2004). Psychological impact of genetic counseling for familial cancer: a systematic review and meta-analysis. J Natl Cancer Inst.

[29] Walter F, Prevost A, Birt L, Grehan N, Restarick K, Morris H, et al. Development and evaluation of a brief self-completed family history screening tool for common chronic disease prevention in primary care. Br J Gen Pract. 2013;63(611):e393–e400. [cited 2017 Aug 17]. Available from: 10.3399/bjgp13X668186. PMC3662456 23735410

[30] Marteau TM, Bekker H. The development of a six-item short-form of the state scale of the Spielberger State-Trait Anxiety Inventory (STAI). Br J Clin Psychol [Internet]. 1992 Sep 1 [cited 2017 Dec 7];31(3):301–6. Available from: http://doi.wiley.com/10.1111/j.2044-8260.1992.tb00997.x. 10.1111/j.2044-8260.1992.tb00997.x 1393159

[31] Lerman C, Trock B, Rimer BK, Jepson C, Brody D, Boyce A. Psychological side effects of breast cancer screening. Heal Psychol. 1991;10(4):259–67. [cited 2017 Aug 17]. Available from: https://www.ncbi.nlm.nih.gov/pubmed/1915212. 10.1037//0278-6133.10.4.259 1915212

[32] Emery J, Morris H, Goodchild R, Fanshawe T, Prevost AT, Bobrow M (2007). The GRAIDS trial: a cluster randomised controlled trial of computer decision support for the management of familial cancer risk in primary care. Br J Cancer.

[33] Connor M, Sparks P. Theory of Planned Behaviour and Health Behaviour. In: Connor M, Norman P, editors. Predicting Health Behaviour [Internet]. 2nd ed. Maidenhead: Open University Press; 2005. p. 170–222. [cited 2017 Jul 20].

[34] Australian Government Department of Health and Ageing. Medicare benefits schedule. Canberra, ACT: Australian Government; 2017.

[35] Emery JD, Reid G, Prevost AT, Ravine D, Walter FM (2014). Development and validation of a family history screening questionnaire in Australian primary care. Ann Fam Med.

[36] Australian Institute of Health and Welfare. Analysis of bowel cancer outcomes for the National Bowel Cancer Screening Program [Internet]. [cited 2017 Aug 29]. 62 p. Available from: http://www.aihw.gov.au/publication-detail/?id=60129549725.

[37] Viiala CH, Zimmerman M, Cullen DJE, Hoffman NE (2003). Complication rates of colonoscopy in an Australian teaching hospital environment. Intern Med J.

[38] StataCorp. Stata statistical software: release 15. College Station, TX: StataCorp LLC; 2017. [cited 2017 Dec 6]. Available from: www.stata.com

[39] White IR, Carpenter J, Horton NJ (2012). Including all individuals is not enough: lessons for intention-to-treat analysis. Clin Trials.

[40] Harris PA, Taylor R, Thielke R, Payne J, Gonzalez N, Conde JG (2009). Research electronic data capture (REDCap)—a metadata-driven methodology and workflow process for providing translational research informatics support. J Biomed Inform.

[41] Reid GT, Walter FM, Emery JD. A qualitative evaluation of the psychosocial impact of family history screening in Australian primary care. [cited 2017 Aug 30]; Available from: https://link.springer.com/article/10.1007/s10897-014-9772-x. 10.1007/s10897-014-9772-x 25273950

[42] Thorpe KE, Zwarenstein M, Oxman AD, Treweek S, Furberg CD, Altman DG, et al. A pragmatic-explanatory continuum indicator summary (PRECIS): a tool to help trial designers. CMAJ. 2009;180(10):E47–E57. [cited 2017 Aug 30]. Available from: http://www.ncbi.nlm.nih.gov/pubmed/19372436. 10.1503/cmaj.090523 PMC2679824 19372436

[43] Puffer S, Torgerson D, Watson J (2003). Evidence for risk of bias in cluster randomised trials: review of recent trials published in three general medical journals. BMJ.

[44] Murray SR, Murchie P, Campbell N, Walter FM, Mazza D, Habgood E, et al. Protocol for the CHEST Australia Trial: a phase II randomised controlled trial of an intervention to reduce time-to-consult with symptoms of lung cancer. BMJ Open. 2015;5 [cited 2017 Aug 30]. Available from: 10.1136/bmjopen-2015-008046 10.1136/bmjopen-2015-008046 PMC4442170 25986641

