# Supplementary material for: The use of a risk assessment and decision support tool (CRISP) compared with usual care in general practice to increase risk-stratified colorectal cancer screening: study protocol for a randomised controlled trial
Source: Trials. 2018 Jul 25;19:397. doi: 10.1186/s13063-018-2764-7 (PMC6060496; doi:10.1186/s13063-018-2764-7)
Supplement: Supplementary file 2 — Informed consent materials. Plain language statements and consent forms. (PDF 376 kb) [file 13063_2018_2764_MOESM2_ESM.pdf]

Who are we?

The CRE is made up of a highly motivated team of cancer researchers including epidemiologists, primary care researchers and GPs from the University of Melbourne, Royal Melbourne Hospital, Peter MacCallum Cancer Centre, The Cancer Council Victoria, Western Health and the Victorian Comprehensive Cancer Centre. The researchers involved in this study include: Prof Jon Emery, A/Prof Marie Pirotta, Dr Jennifer Walker, Ms Sibel Saya on behalf of the CRISP investigators. [Full list of investigators on our website - www.crisp.org.au/about-us].

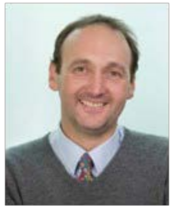

Professor Jon Emery

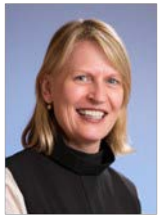

Dr Jennifer Walker

THE UNIVERSITY OF MELBOURNE

Further information and contact details

Please contact Dr Jennifer Walker or Professor Jon Emery if you have any questions.

Dr Jennifer Walker
Research Fellow
Department of General Practice
Victorian Comprehensive Cancer Centre
University of Melbourne
Level 10, 305 Grattan Street, Uni of Melbourne 3010
T: (03) 8559 7048
M: 0408 510 248
E: walker@unimelb.edu.au

Professor Jon Emery
Herman Professor of Primary Care Cancer Research, University of Melbourne
Department of General Practice
Victorian Comprehensive Cancer Centre
University of Melbourne
Level 10, 305 Grattan Street, Uni of Melbourne 3010
T: (03) 8559 7044
E: jon.emery@unimelb.edu.au

What if there is a problem? If you have concerns about the conduct of this research project, please contact the Manager, Human Research Ethics Committee, The University of Melbourne on (03) 8344 2071 or fax (03) 9347 6739.

Ethics ID: 1647804.5
(Patient Plain Language Statement Version 5, 20_March_2018)

Thank you for your involvement in this important research.

CRE CRC Centre of Research Excellence: Optimising Screening for Colorectal Cancer

The *CRISP* trial: assessing bowel cancer risk in general practice.

PATIENT PLAIN LANGUAGE STATEMENT

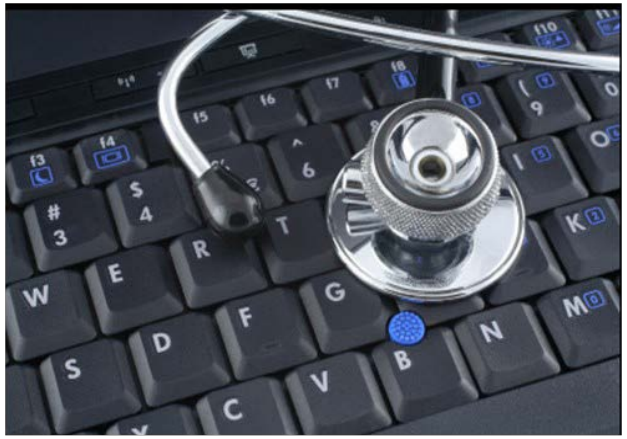

Laptop and stethoscope by jfcherry CC BY-SA 2

PARTICIPANT PLAIN LANGUAGE STATEMENT

This Plain Language Statement provides important information about this research project including the procedures involved. If you decide to participate in this research study, you will be asked to sign a consent form and by signing it you will be agreeing that:

You understand this Plain Language Statement

You consent to participate in the research processes as described

You consent to the use of your personal information as described

What is the purpose of this research?

In this study we are testing whether using a computer to estimate bowel cancer risk will help people make better decisions about screening for bowel cancer. A research project officer will work with you to complete the computer program (called the 'CRISP' tool) and then give you your risk of developing bowel cancer and how to best test for it. We are looking at different ways of providing information about bowel cancer risk to patients and whether a computer program aimed at providing advice about cancer screening could help. The research is led by Professor Jon Emery and is part of the 'NHMRC funded Centre for Research Excellence for Reducing the Burden of Colorectal Cancer by Optimising Screening: Evidence to Clinical Practice' [APP1042021] and the Victorian Cancer Agency [HSR15019].

Why have I been invited?

We are asking all patients attending this practice who are between 50 and 74 years old with a current Medicare or Veterans' Health card, whose doctor has consented to be involved.

Your participation is voluntary and you can withdraw at any stage by contacting the researchers on the details overleaf. Any unprocessed information you have provided will be destroyed. Withdrawing from the study will not affect your care in any way.

What will you be asked to do?

If you agree to take part and sign a consent form, you will be placed in one of two groups of the study (group A or group B) at random. Randomisation means that you are put into a group by chance, like the toss of a coin. Neither you nor your doctor can choose the group you will be in.

Group A will use a computer program which asks you questions about you and your family history. Group B will not use the computer program. The researcher will give you some information about cancer prevention and screening. Both groups will be provided with information about cancer prevention from the Cancer Council Victoria. We will be videotaping some of the consultations, please let the research team know if you DO NOT wish to be recorded.

All participants will be asked to fill in a survey about their health including family history of cancer and lifestyle. We will send you 3 more surveys over the next 12 months. A text message reminder to undertake the recommended screening will also be sent at 1 month. Some participants will be interviewed over the telephone about their experience of being involved in the study and if they choose they can complete the survey over the phone. If you are contacted to be interviewed, please note that the interview is optional and will be recorded if you agree, to be used for research purposes. We also ask your consent to look at some other of your medical records (Medicare, National Bowel Cancer Screening Program, Department of Health and Human Services, Victoria and GP). This will allow us to check details of any bowel cancer screening that you will have during the next 5 years and the previous 4 ½ years.

You will be asked to fill out a consent form authorising the study access to your complete Medicare data as outlined on the back of the consent form. Medicare collects information on your doctor visits and the associated costs. The consent form is sent securely to the Commonwealth Department of Human Services who holds this information confidentially.

It is anticipated that this will take 15-20 minutes of your time today; later the telephone interviews will take a maximum of 20 minutes each. You will not miss your appointment with your doctor and it is important that you discuss any concerns with your GP when you have your consultation.

How will this study affect me?

Being involved in this study, you might receive personal advice about testing (and in some cases an at-home Faecal Occult Blood Test kit) for bowel cancer. This advice is based on either the most up-to-date National Health and Medical Research Council guidelines or the computer risk tool (CRISP), whichever is more conservative. Any screening investigations recommended as a result of being involved in this study this will be under the care of your GP and/or medical specialist.

Will my taking part in this study be kept confidential?

All information you provide will remain strictly confidential, and you will be given a unique ID for any identifying information. All data will be stored securely on University of Melbourne password- protected computers, which are stored within locked facilities. Data will be disposed of after 5 years according to the University of Melbourne guidelines which includes shredding of all study documents including your study and Medicare consent form and deleting any data from hard drives and servers.

Involvement of your General Practitioner (GP)

Your GP has consented to being involved in this study. If you agree to take part we will let them know of your involvement with your permission.

THE UNIVERSITY OF MELBOURNE

ID: _ _/_ _
Date: _ _/_ _/_ _

Department of General Practice, University of Melbourne

CONSENT FORM FOR PATIENTS

Project title: The CRISP trial: assessing bowel cancer risk in general practice.

Name of participant: ____

Name of investigator(s):
Prof Jon Emery, A/Prof Marie Pirotta, Dr Jennifer Walker, Ms Sibel Saya, Ms Jasmeen Oberoi, Ms Kitty Novy, and Ms Kristi Milley on behalf of the CRISP investigators

1. I consent to take part in this research project, the details of which have been explained to me, and I have been provided with a written participant information form (Participant Information Form Version 5, 20_March_2018) to keep.
2. I understand that after I sign and return this consent form it will be retained by the researcher.
3. I understand that my participation will involve a computer based questionnaire (if in the intervention group) and I agree that the researchers may use the results as described in the participant information form.
4. I allow the release of my medical information from my medical records including general practice, Medicare, the National Bowel Cancer Screening Program and Victorian Hospital data that relates to previous screening for bowel cancer and during the next 5 years.
5. I acknowledge that:
    a. The possible effects of participating in the questionnaire and computer based program (if in the intervention group) have been explained to my satisfaction;
    b. I understand that my involvement in this study is entirely voluntary;
    c. I have been informed that I am free to withdraw from the project at any time without explanation or prejudice and to withdraw any unprocessed data I have provided;
    d. The project is for the purposes of research;
    e. I have been informed that the confidentiality of the information I provide will be safeguarded subject to any legal requirements;
    f. I have been informed that my consent form and information will be stored securely at the University of Melbourne and destroyed after 5 years;
    g. My name will not be identified in any publications arising from the research;
    h. I have been informed that a copy of the research findings will be forwarded to me, should I request it at the completion of the study;
    i. In accordance with the law of Victoria, I understand that it is possible for data to be subject to subpoena, or freedom of information request.

THE UNIVERSITY OF MELBOURNE

6. I understand my consultation might be video-recorded and that this is voluntary. The video recording will only be used for research purposes and my choice not to be video-recorded will not be disclosed to anyone except the research team.

Please tick one box: ☐ I consent to be video-recorded ☐ I do not consent to be video-recorded

7. I consent to the research team contacting me for a follow up telephone interview about my experience of being involved in the study. The interview will be recorded but is optional. The number I can be contacted on is below:

Contact number: ____

Preferred method for follow up surveys:

◯ online survey

(email address) ____

◯ postal survey

(mailing address)

Name: ____

Street: ____

Suburb: ____

State: ____________________ Postcode: ______________________________

Participant signature: ____ Date: ____________

Researcher's Name (printed) __________________________________ Date: ____________

Signature: ____ Date: ____________

Please complete the following pages to allow the release of your Medicare data, National Bowel Cancer Screening data and data from the Department of Health and Human Services, Victoria.

We will only access data that relates to screening for bowel cancer during the next 5 years and the previous 4 ½ years.

This study has been funded by the Australian National Health and Medical Research Council [APP1042021] and the Victorian Cancer Agency [HSR15019].

Participant ID:

PARTICIPANT CONSENT FORM

Consent to release of Medicare claims information for the purposes of **The CRISP trial: assessing risk of bowel cancer in general practice.**

Important Information
Complete this form to request the release of personal Medicare claims information to **The CRISP trial: assessing risk of bowel cancer in general practice.**

Any changes to this form must be initialled by the signatory. Incomplete forms may result in the study not being provided with my information.

By signing this form, I acknowledge that I have been fully informed and have been provided with information about this study. I have been given an opportunity to ask questions and understand the possibilities of disclosures of my personal information.

PARTICIPANT DETAILS

1. Mr ☐ Mrs ☐ Miss ☐ Ms ☐ Other ☐

Family name: ______________________________ First given name: ________________________

Other given name (s): ________________________

Date of birth: DD / MM / YYYY

2. Medicare card number: ____________________

3. Permanent address: ____

Postal address (if different to above): ____

AUTHORISATION

4. I authorise Department of Human Services to provide my:

☐ Medicare claims history

for the period* DD / MM / YYYY to: DD / MM / YYYY to the CRISP trial.

*Note: The Department of Human Services can only extract 4 ½ years of data (prior to the date of extraction). The consent period above may result in multiple extractions.

DECLARATION

I declare that the information on this form is true and correct.

5. Signed: ____________________ (participant's signature) Dated: DD / MM / YYYY OR

6. Signed by: ____________________ (full name) ________________ (signature) on behalf of participant

Dated: DD / MM / YYYY

☐ Power of attorney* ☐ Guardianship order*

*Please attach supporting evidence

APP 5 – PRIVACY NOTICE

Your personal information is protected by law, including the Privacy Act 1988, and is collected by the Australian Government Department of Human Services. The collection of your personal information by the department is necessary for administering requests for statistical and other data.

Your information may be used by the department or given to other parties for the purposes of research, investigation or where you have agreed or it is required or authorised by law.

You can get more information about the way in which the Department of Human Services will manage your personal information, including our privacy policy at humanservices.gov.au/privacy or by requesting a copy from the department.

Power of attorney – a power of attorney is a document that appoints a person to act on behalf of another person who grants that power. In particular, an enduring power of attorney allows the person to act on behalf of another person even when that person has become mentally incapacitated. The powers under a power of attorney may be unlimited or limited to specific acts.

Guardianship order – a guardianship order is an order made by a Guardianship Board/Tribunal that appoints a guardian to make decisions for another person. A Guardianship order may be expressed broadly or limited to particular aspects of the care of another person.

A sample of the information that may be included in your Medicare claims history:

Date of service	Date of processing	Item number	Item description	Provider charge	Schedule Fee	Benefit paid	Patient out of pocket
20/04/09	03/05/09	00023	Level B consultation	$38.30	$34.30	$34.30	$4.00
22/06/09	23/06/09	11700	ECG	$29.50	$29.50	$29.50	

Date of referral	Ordering Provider postcode	Provider derived major speciality	Item category
		General Practitioner	1
20/04/09	2302	Cardiologist	2

PARTICIPANT CONSENT FORM- Previous 4 ½ Years

Consent to release of data from the Victorian Admitted Episodes Dataset (VAED) related to colonoscopy or bowel cancer for the purposes of **The CRISP trial: assessing risk of bowel cancer in general practice.**

Important Information

Complete this form to request the release of personal VAED information to **The CRISP trial: assessing risk of bowel cancer in general practice.**

Any changes to this form must be initialled by the signatory. Incomplete forms may result in the study not being provided with my information.

By signing this form, I acknowledge that I have been provided with information about this study. I have been given an opportunity to ask questions and have been fully informed about this study.

PARTICIPANT DETAILS

1. Mr ☐ Mrs ☐ Miss ☐ Ms ☐ Other ☐

Family name: ________________________________ First given name: _____________________

Other given name (s): __________________________

Date of birth: ___/___/_____

2. Medicare card number: ______________________

3. Permanent address: ____

Postal address (if different to above): ____

AUTHORISATION

4. I authorise the Department of Health and Human Services to provide my:
medical details regarding hospital visits for the period* ___/___/_____ to: ___/___/_____ to the **The CRISP trial: assessing risk of bowel cancer in general practice**.

*Note: This period cannot exceed 4 ½ years

DECLARATION

I declare that the information on this form is true and correct.

5. Signed: ______________________ (participant's signature)

PARTICIPANT CONSENT FORM- Next 5 Years

Consent to release of data from the Victorian Admitted Episodes Dataset (VAED) related to colonoscopy or bowel cancer for the purposes of **The CRISP trial: assessing risk of bowel cancer in general practice.**

Important Information

Complete this form to request the release of personal VAED information to **The CRISP trial: assessing risk of bowel cancer in general practice.**

Any changes to this form must be initialled by the signatory. Incomplete forms may result in the study not being provided with my information.

By signing this form, I acknowledge that I have been provided with information about this study. I have been given an opportunity to ask questions and have been fully informed about this study.

PARTICIPANT DETAILS

1. Mr ☐ Mrs ☐ Miss ☐ Ms ☐ Other ☐

Family name: ________________________________ First given name: _____________________

Other given name (s): __________________________

Date of birth: ___/___/_____

2. Medicare card number: ______________________

3. Permanent address: ____

Postal address (if different to above): ____

AUTHORISATION

4. I authorise the Department of Health and Human Services to provide my:
medical details regarding hospital visits for the period* ___/___/_____ to: ___/___/_____ to the **The CRISP trial: assessing risk of bowel cancer in general practice**.

*Note: This period cannot exceed 4 ½ years

DECLARATION

I declare that the information on this form is true and correct.

5. Signed: ______________________ (participant's signature)
