# Supplementary material for: The use of a risk assessment and decision support tool (CRISP) compared with usual care in general practice to increase risk-stratified colorectal cancer screening: study protocol for a randomised controlled trial
Source: Trials. 2018 Jul 25;19:397. doi: 10.1186/s13063-018-2764-7 (PMC6060496; doi:10.1186/s13063-018-2764-7)
Supplement: Supplementary file 3 — Additional outcome variables. (DOCX 17 kb) [file 13063_2018_2764_MOESM3_ESM.docx]

Cancer Risk Perception

This will be assessed using a combination of measures which have been validated from our previously published systematic reviews and primary research on colorectal cancer risk.(1, 2)

(a) participants will be asked to provide a numerical value on a scale of 0 to 100 of their chances of getting bowel [“*If you had to put a figure on it, what would you say were your chances of getting bowel cancer at some time in your life*?”]

(b) participants will also provide a comparative measure on a 5-point scale of their chances of developing bowel cancer compared to average person of their age (“*How likely are you to develop bowel cancer compared to other people of your age*?” 1, much less likely; 3, about the same; 5, much more likely).

The State-Trait Anxiety Inventory (STAI)

The six-item short-form of the STAI will be used to measure the current state of anxiety. Responses will assess the intensity of general anxiety feelings “right now at this moment” with options: 1) not at all, 2) somewhat, 3) moderately, and 4) very much. This brief form of STAI questionnaire has reliability and validity comparable to the full form of the STAI. (3)

Cancer Worry Scale (CWS)

Participants will be asked about their thoughts about their own personal risk of developing bowel cancer at baseline, 1, 6 and 12 months. This will be assessed with item measure that includes items assessing frequency and its extent “*How often do you worry about developing bowel cancer?” and “How much of a problem is worrying about bowel cancer to you*”. Participants will also the rate the impact of cancer worrying (if any) on their moods and ability to perform their daily activities rated on a 4-point scale “*How often have thoughts about your chances of getting bowel cancer affected your mood*?”. These measures are scored on a 4-point scale as 1) not at all or rarely; 2) sometimes; 3) often; and 4) almost all the time. This measure has been previously well validated for the frequency of breast cancer worry and the impact of worrying on mood and ability to perform daily activities.(4,5)

CRC screening behaviour/ intentions

Based on the Theory of Planned Behaviour, it is known that intention to perform a behaviour is a strong predictor of performing the behaviour.(2,6) Being screened for CRC will be assessed at 1,6 and 12-month follow-up asking whether the participant had done any bowel cancer screening test in the past month with options of yes or no. The intention to be screened for CRC will be measured in the follow up surveys at the time periods stipulated above asking whether the participant intends to screen for bowel cancer in the next three months on a 5-point scale from “strongly disagree” to “strongly agree”. This will allow us to examine the effect the of CRISP intervention on CRC screening intentions and behaviour. Screening behaviour will be assessed 12 months and again at 5 years from baseline in both intervention and control groups using GP medical records, Medicare data and VAED information.

1. Braithwaite D, Emery J, Walter F, Prevost AT, Sutton S. Psychological impact of genetic counseling for familial cancer: a systematic review and meta-analysis. J Natl Cancer Inst. 2004;96(2):122–33.

2. Walter F, Prevost A, Birt L, Grehan N, Restarick K, Morris H, et al. Development and evaluation of a brief self-completed family history screening tool for common chronic disease prevention in primary care. Br J Gen Pract. 2013;63(611):e393–e400. [cited 2017 Aug 17]. Available from: <https://doi.org/10.3399/bjgp13X668186>.

3. Marteau TM, Bekker H. The development of a six-item short-form of the state scale of the Spielberger State-Trait Anxiety Inventory (STAI). Br J Clin Psychol [Internet]. 1992 Sep 1 [cited 2017 Dec 7];31(3):301–6. Available from: <http://doi.wiley.com/10.1111/j.2044-8260.1992.tb00997.x>

4. Lerman C, Trock B, Rimer BK, Jepson C, Brody D, Boyce A. Psychological side effects of breast cancer screening. Heal Psychol. 1991;10(4):259–67. [cited 2017 Aug 17]. Available from: https://www.ncbi.nlm.nih.gov/pubmed/1915212.

5. Emery J, Morris H, Goodchild R, Fanshawe T, Prevost AT, Bobrow M, et al. The GRAIDS trial: a cluster randomised controlled trial of computer decision support for the management of familial cancer risk in primary care. Br J Cancer. 2007;97(4):486–93.

6. Connor M, Sparks P. Theory of Planned Behaviour and Health Behaviour. In: Connor M, Norman P, editors. Predicting Health Behaviour [Internet]. 2nd ed. Maidenhead: Open University Press; 2005. p. 170–222. [cited 2017 Jul 20].
